# Specific Antibodies for the Detection of *Alternaria* Allergens and the Identification of Cross-Reactive Antigens in Other Fungi

**DOI:** 10.1159/000449415

**Published:** 2016-10-26

**Authors:** Teresa E. Twaroch, Mirela Curin, Katja Sterflinger, Margit Focke-Tejkl, Ines Swoboda, Rudolf Valenta

**Affiliations:** a Christian Doppler Laboratory for Allergy Research, Division of Immunopathology, Department of Pathophysiology and Allergy Research, Center for Pathophysiology, Infectiology and Immunology, Medical University of Vienna; b Department for Biotechnology, University of Natural Resources and Applied Life Sciences, Vienna, Austria

**Keywords:** Allergens, Peptide-specific antibodies, Cross-reactive antigens, *Alternaria alternata*

## Abstract

**Background:**

The mould *Alternaria alternata* is an important source of respiratory allergens. *A. alternata* extracts show great variations regarding allergenic potency. The aim of this study was to generate antibody probes specific for important *Alternaria* allergens and to use them to study allergen expression, depending on different culture conditions, as well as to search for cross-reactive allergens in other mould species.

**Methods:**

Synthetic peptides from antigenic regions of *A. alternata* allergens (Alt a 1, Alt a 2, Alt a 3, Alt a 6 and Alt a 8) were used to raise highly specific rabbit antibodies. These antibodies and IgE from allergic patients were used to detect allergens by immunoblotting in extracts of 4 *A. alternata* strains grown under varying culturing conditions, in commercial skin-prick extracts and in closely (*Cladosporium herbarum* and *Aureobasidium pullulans*) or distantly related (*Aspergillus niger* and *Penicillium chrysogenum*) mould species.

**Results:**

There was a wide variation of expression of the individual *A. Alternata* allergens, depending on the strain and culture conditions, but the antibody probes allowed us to distinguish strains and culture conditions with low and high allergen expression. In the commercial skin-prick solutions, varying levels of Alt a 1 were found, but no other allergens were detectable. Alt a 1 was identified as species-specific *A. Alternata* allergen, whereas Alt a 3, 6- and Alt a 8-cross-reactive antigens were found in *C. herbarum* and/or *A. pullulans*.

**Conclusions and Clinical Relevance:**

Peptide-specific antibodies are useful to analyze diagnostic and therapeutic mould extracts, to study the presence of *A. Alternata* allergens in biological samples and to search for cross-reactive allergens in other mould species.

## Introduction

Recent studies demonstrate that moulds are an important allergen source that has been underestimated for a long time [1, 2]. Moulds have also been shown to have immunomodulatory capacity [3–5]. Investigations on allergen content, allergenicity and immunogenicity have been hampered by the multiplicity of mould species in our environment [6], variations between strains of the same species [7–12] and the tendency of moulds to mutate frequently [13]. Several other factors hamper the production of diagnostic and therapeutic mould allergen extracts. These factors include the selection of suitable mould strains, the choice of appropriate culture conditions and adequate manufacturing protocols [11, 12, 14–16].

One of the best-studied mould species is the genus *Alternaria*, which represents an important inducer of allergy and asthma [17, 18]. *Alternaria* also affects the innate immune system and may enhance inflammation caused by unrelated allergens (e.g. grass pollen) [19, 20].

Early studies revealed that *Alternaria* extracts differ regarding their protein, carbohydrate and allergenic potency as well as in their enzymatic activity [10–12]. Studies also indicated the importance of culturing and extraction conditions on these parameters [7–12, 21]. However, information on protein or carbohydrate content does not enable conclusions to be drawn regarding the presence of individual allergens and allergen composition. The recognition by allergen-specific antibodies and, in particular, antibody probes specific for the protein backbone of the allergen, is not affected by cross-reactive carbohydrates coupled to various allergens [22]. These antibodies are therefore useful tools for the detection of allergens in natural sources of allergens and their extracts. Monoclonal allergen-specific antibodies may recognize only certain allergen isoforms or certain allergen modifications; it has therefore been argued that oligoclonal antibody probes may be better tools for allergen identification and extract standardization [23].

We thus generated peptide-specific antibodies for important allergens of *A. Alternata* (Alt a 1, Alt a 2, Alt a 3, Alt a 6 and Alt a 8) to analyze the expression of these allergens in different *A. Alternata* strains and under varying culture conditions as well as in commercial extracts for diagnostic skin testing*.* Antibodies were also used to search for cross-reactive antigens in other mould species (*C. herbarum*, *A. pullulans*, *A. niger* and *P. chrysogenum*).

## Methods

### Sera from Allergic Patients

Eight patients from Austria suffering from allergic rhinitis, conjunctivitis and/or asthma due to *A. Alternata* sensitization were diagnosed based on a positive case history and the presence of specific IgE antibody levels >3.5 kU_A_/l to *A. alternata* (corresponding to RAST ≥3) as determined by CAP-FEIA (Phadia, Uppsala, Sweden). Residual serum samples from routine diagnostic procedures were tested in an anonymized manner with approval from the ethics committee of the Medical University of Vienna (EkWr: 565/2007).

### Cultivation of Mould Strains and Extract Preparation

*A. alternata* strains (CBS 103.33 and CBS 795.72), *C. herbarum* (CBS 134.31), *A. niger* (CBS 113.46) and *P. chrysogenum* (CBS 194.46) were obtained from the Fungal Biodiversity Center (Utrecht, The Netherlands). Two *A. alternata* strains (ATCC 46582 and ATCC 96154) were purchased from the American Type Culture Collection (Manassas, Va., USA). The black yeast *A. pullulans* was provided by the Austrian Center of Biological Resources and Applied Mycology (BOKU, Vienna, Austria).

The lyophilized moulds were resuspended in sterile water and cultivated on Sabouraud glucose agar plates (4% glucose, 1% peptone and 1.5% agar-agar), Czapek-Dox agar plates (3% w/v sucrose, 0.3% w/v sodium nitrate, 0.05% w/v magnesium sulfate, 0.05 w/v potassium chloride, 0.001% w/v iron(II)sulfate, 0.1% w/v dipotassium hydrogen phosphate and 1.3% w/v agar-agar) or Malt extract agar plates (2% glucose, 2% maltose, 0.1% peptone and 1.5% agar-agar all w/v) at 28 ° C. After 1, 2 or 4 weeks of growth, fungal mats consisting of spores and mycelium (which was referred as tissue because it also includes spores) were detached from the agar plates using a sterile scalpel, and then ground in liquid nitrogen to a fine powder. The powder was suspended in extraction buffer containing 50 mM sodium bicarbonate (pH 8.6), 150 mM sodium chloride, 2 mM PMSF (phenylmethanesulphonylfluoride), 2 mM EDTA (ethylenediaminetetraacetic acid) and a protease inhibitor mix (Sigma Aldrich, St. Louis, Mo., USA), and then homogenized using an ultraturrax (Stauffen, Germany). Insoluble particles were removed by centrifugation at 4,500 *g* for 20 min at 4 ° C and the extracts were stored at −20 ° C until use. The protein concentration was determined by the Bio-Rad Protein Assay (Bio-Rad Laboratories, Richmond, Calif., USA), using BSA as standard.

### Alternaria Skin-Prick Solutions

*Alternaria* skin-prick solutions were purchased from 5 European manufacturers: (1) Allergopharma 400: *Alternaria tenuis* (*A. alternata*), 10,000 SBE/ml, Ch.-B.: U9004619-AT; (2) ALK Abello B318-L402: *A. tenuis*, 30 HEP, 25 μg Alt a 1/ml, Ch.-B.: D1022; (3) Bencard 1100–030–043: *A. alternata*, 10,000 DU/ml, Ch.-B.: A12217NT; (4) HAL 20–01: *A. alternata*, 5,000 AU/ml, Ch.-B.: B1913045; (5) Alyostal 400: *Alternaria*, 1,000 IC/ml, Ch.-B.: 96176. The protein concentration of the skin-prick solutions was determined after dialysis against distilled water using the Bio-Rad protein assay. Changes in volume caused by dialysis were recorded for each extract and corrected for all subsequent analyses.

### Generation of Allergen-Specific Antibody Probes

ProtScale bioinformatics tool from the ExPASY server was used to predict peptides from surface-exposed regions with high antigenicity (http://web.expasy.org/protscale) [24, 25] of Alt a 1 (aa 19–52, APLESRQDTASCPVTTEGDYVWKISEFYGRKPEG), Alt a 2 (aa 10–35, DNIFRSLSKEDPDYSRNIEGQVIRLH), Alt a 3 (aa 85–110, LDDNQTATKDEYESQQKELEGVANPI), Alt a 6 (aa 250–275, SEFYKADEKKYDLDFKNPDSDKSKWL) and Alt a 8 (aa 146–173, FRERKTGSLVITSSMSGHIANFPQEQAS). Peptides were synthesized on an Applied Biosystems peptide synthesizer, Model 433A (Foster City, Calif., USA), as previously described [26]. Peptides were identified by mass spectrometry and purified to >90% purity by preparative HPLC (piChem, Graz, Austria). Allergen-derived peptides were coupled to KLH (Keyhole limpet hemocyanin; Pierce, Rockford, Ill., USA) and rabbits were immunized 3 times at 4-week intervals with 200 μg of the KLH-conjugated peptides per injection, using Freund’s complete and incomplete adjuvant (Charles River, Kisslegg, Germany) [26]. Serum samples were obtained before immunization (preimmune sera) and in 4-week intervals after immunization, and then stored at −20 ° C.

### Detection of Alternaria Allergens with Patients’ IgE or Rabbit Antibodies

Protein extracts from *A. alternata*, *C. herbarum*, *A. pullulans*, *A. niger* or *P. chrysogenum* (approx. 80 μg/cm gel) were separated on a 15% preparative SDS polyacrylamide gel [27] under reducing conditions and blotted onto nitrocellulose membranes (Protran^®^ nitrocellulose transfer membrane, Whatman^®^, Schleicher & Schuell, Dassel, Germany). Nitrocellulose strips were cut from the membranes, blocked in PBST (PBS, 0.5% v/v Tween 20) and exposed overnight at 4 ° C with 1:10 diluted patients’ sera, with 1:5,000 diluted rabbit antisera or with a mixture of the preimmune sera. After washing with PBST, bound antibodies were either detected with ^125^I-labelled anti-human IgE (1:15) (IBL, Hamburg, Germany) or ^125^I-labelled goat anti-rabbit IgG (1:3,000) (Perkin Elmer, Waltham, Mass., USA) and visualized by autoradiography.

## Results

### The Expression Level of Alternaria Allergens Varies in Different Strains

The determination of total protein contents showed that comparable levels of protein could be extracted from the strains CBS 103.33 and ATCC 96154 (approx. 8.5 mg/5 g tissue) after 1 week of growth on Sabouraud glucose agar (data not shown). In the extract from strain ATCC 45682, the protein concentration was only half of this amount and approximately one quarter in the case of strain CBS 795.72 (data not shown).

**Fig. 1:**
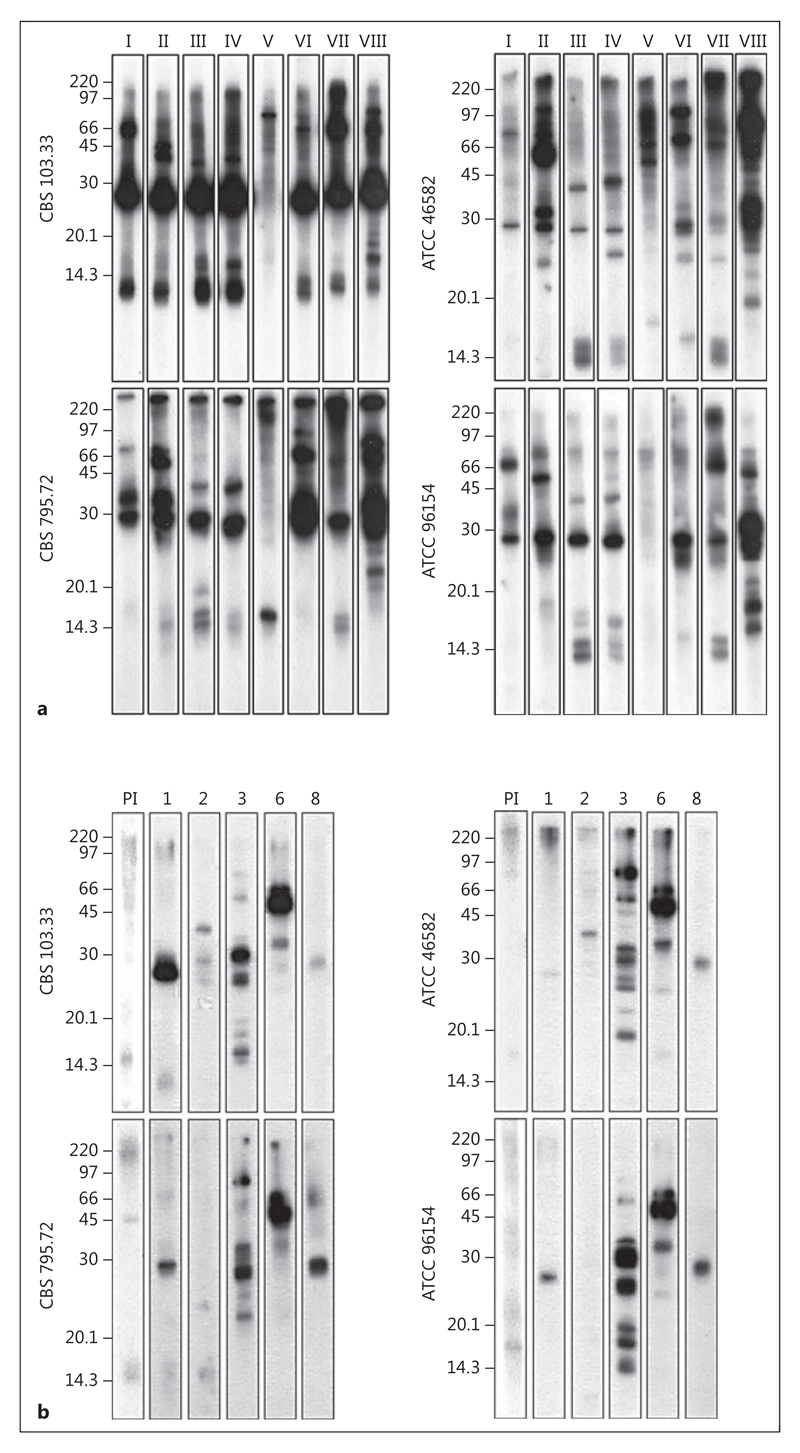
Variability of allergen expression in 4 different *A. alternata* strains. Nitrocellulose-blotted extracts of 4 *A. alternata* strains (CBS 103.33, CBS 795.72, ATCC 48562 and ATCC 96154) were incubated with sera from 8 *Alternaria*-allergic patients (I–VIII) (**a**) or with peptide-specific rabbit antibodies (**b**) (lanes 1, 2, 3, 6 and 8: αAlt a 1, αAlt a 2, αAlt a 3, αAlt a 6 and αAlt a 8; lane PI: mixture of rabbit preimmune sera). Molecular weights (kDa) are indicated in the left margin.

Next, the allergen composition was investigated in extracts from the 4 strains. Nitrocellulose-blotted *A. alternata* extracts were probed with sera from 8 *Alternaria*-allergic patients (fig. 1a) or with antibody probes specific for Alt a 1, Alt a 2, Alt a 3, Alt a 6 and Alt a 8 (fig. 1b). Allergic patients’ IgE antibodies reacted with a broad spectrum of bands ranging from 10 to 200 kDa, which showed a considerable variation between the different strains. Screening with the allergen-specific antibody probes revealed the presence of Alt a 1 (30 kDa), Alt a 3 (14–85 kDa), Alt a 6 (47 kDa) and Alt a 8 (29 kDa) whereas Alt a 2 was not detected in any of the 4 extracts. The expression levels of the different allergens varied between the extracts, especially for Alt a 1, which was expressed in much higher amounts in the strain CBS 103.33. Alt a 6 was detectable in comparable amounts in each of the strains whereas the strongest signal for Alt a 8 was observed in the strain CBS 795.72 and only weak reactivity in strains CBS 103.33 and ATCC 46582. Several Alt a 3 bands were found which were smaller than the expected 85 kDa band. This may be due to self-degradation processes which have been described in the case of heat shock proteins [28].

### Variation of Allergen Expression depending on Growth Conditions and Time of Growth

**Fig. 2:**
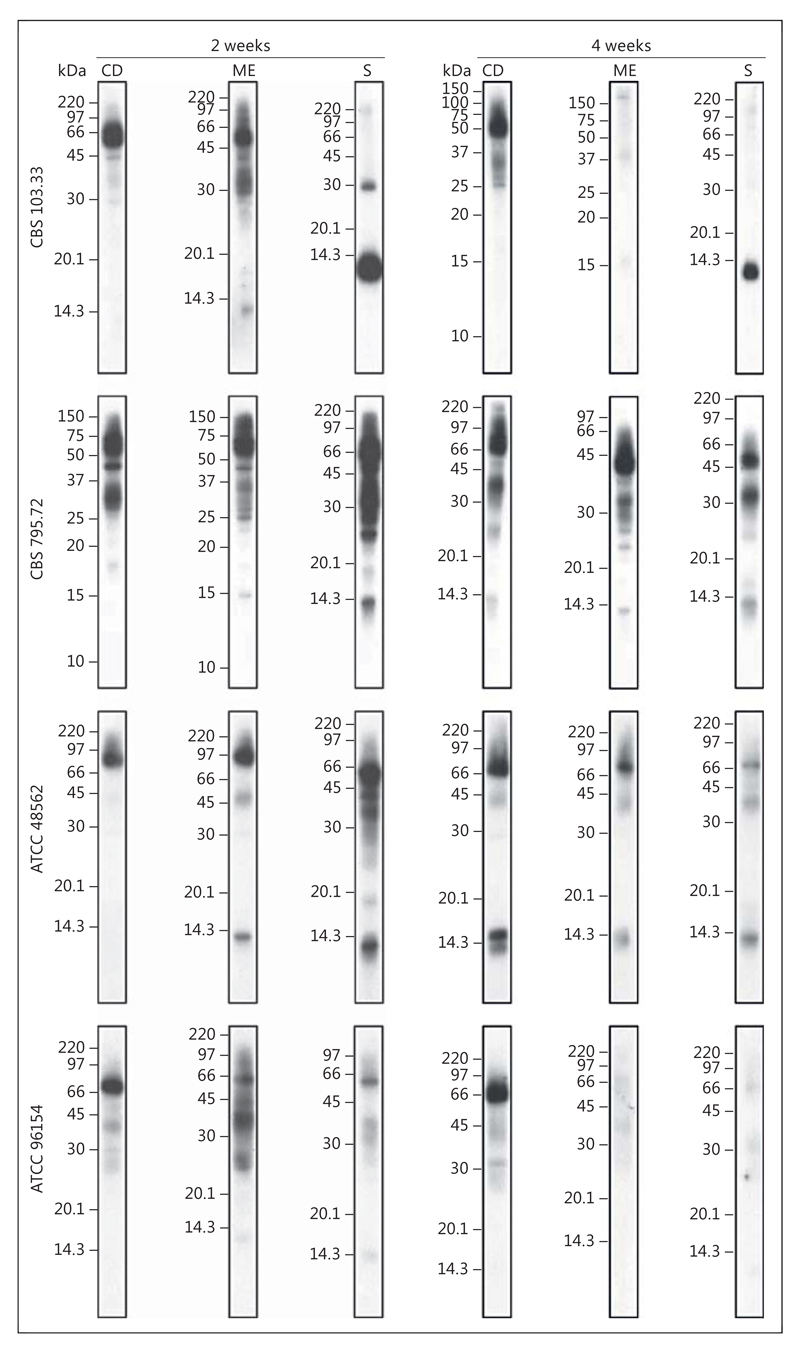
Influence of culture conditions on the expression of *Alternaria* allergens. Extracts of 4 *A. alternata* strains, grown for 2 or 4 weeks on 3 different culturing media, i.e. Czapek-Dox (CD), Malt extract (ME) or Sabouraud (S) agar plates, were blotted onto nitrocellulose and incubated with a serum pool from 3 *Alternaria*-allergic patients. Molecular weights are indicated in the left margin.

We investigated the influence of different growth media (Sabouraud, Malt extract and Czapek-Dox agar) which are commonly used for mould cultivation. These 3 media vary in the composition of nutrients. Furthermore, we studied different growth times (i.e. 2 and 4 weeks; fig. 2).

The total protein contents of the extracts showed very broad variations depending on strain and medium, ranging from 452 μg (ATCC 46582, Malt extract agar, 4 weeks) to 7.5 mg per 5 g of mould tissue (CBS 795.72, Sabouraud glucose agar, 2 weeks). The patterns of IgE-reactive bands in the extracts from each strain varied strongly depending on the culture medium used. For example, a prominent 66-kDa band was detected in CBS 103.33 cultured in Czapek-Dox medium whereas an additional 35-kDa band was found when the strain was cultured in Malt extract and a prominent band at 13-kDa appeared upon cultivation in Sabouraud medium (fig. 2, left upper panel). When the strains were cultivated for 4 weeks, a decrease of IgE-reactive protein bands was often observed in the extracts (fig. 2). Testing of the extracts with allergen-specific antibodies reflected these quantitative differences and visualized qualitative differences regarding allergen contents (table 1). Using certain strains and media, Alt a 1, 3, 6 and 8 could be detected whereas under particular conditions (e.g. strain ATCC 96154, Sabouraud medium), none of these allergens was detected.

### Commercial Alternaria Skin-Prick Solutions Contain Varying Amounts of Alt a 1 and Lack Other Alternaria Allergens

**Fig. 3:**
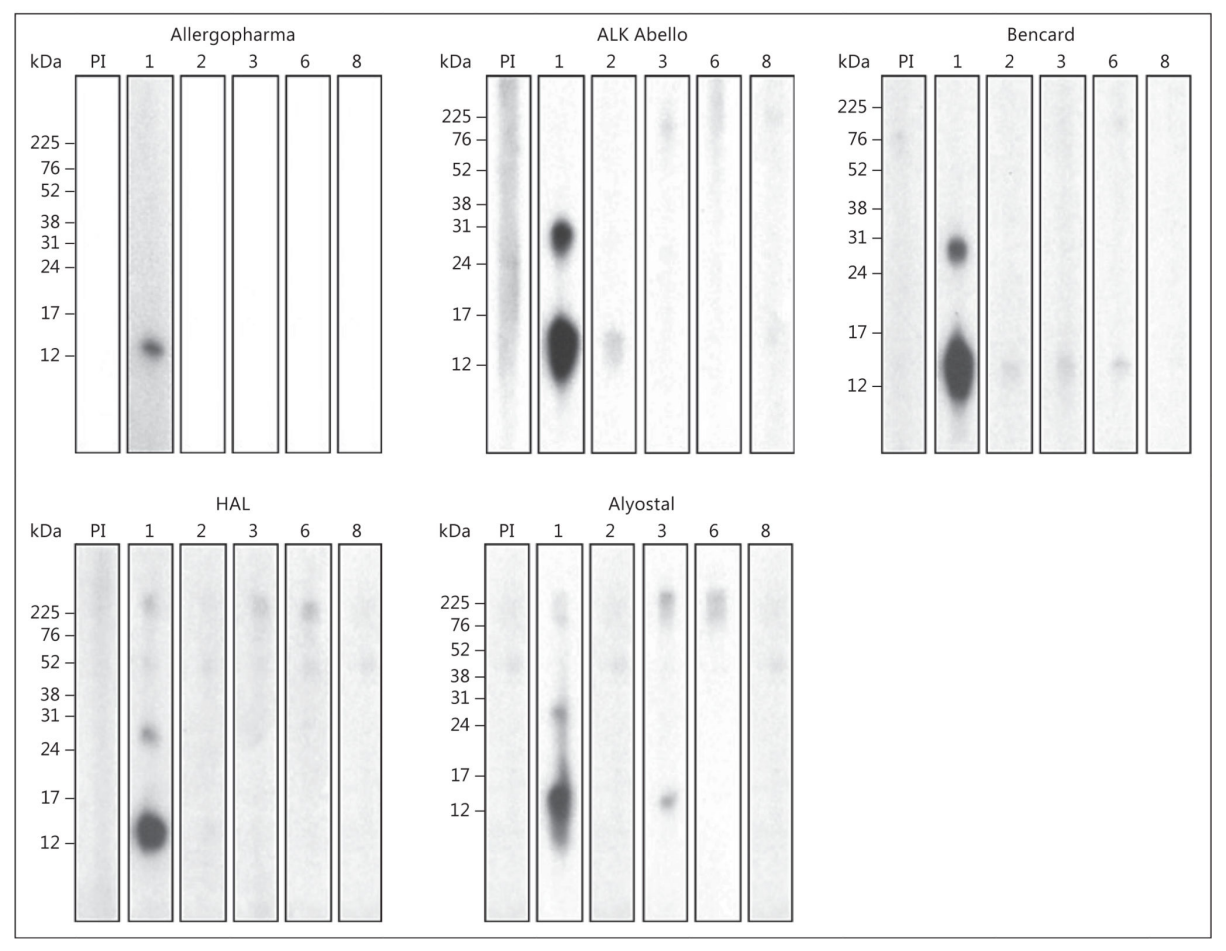
Variations regarding allergen contents in commercially available skin-prick solutions. Nitrocellulose-blotted *Alternaria* skin-prick solutions were screened for the presence of major and minor allergens with specific antibodies (lanes 1, 2, 3, 6 and 8: αAlt a 1, αAlt a 2, αAlt a 3, αAlt a 6 and αAlt a 8) or, for control purposes, with a mixture of preimmune antisera (lane PI). Molecular weights are indicated in the left margin.

The highest total protein concentration was found in the skin-prick extract from Alyostal (441.1 μg/ml), followed by that from HAL (253.1 μg/ml), ALK Abello (187.1 μg/ml), Allergopharma (56 μg/ml) and Bencard (3.1 μg/ml). The total protein concentration of Alyostal was therefore approximately 142-fold higher than the one determined for Bencard. Testing of the extracts with allergen-specific antibodies revealed that the amounts of Alt a 1 were highest in the extracts from Bencard, and ALK, followed by those from HAL and Alyostal and very low in the extract from Allergopharma (fig. 3). None of the other allergens (i.e., Alt a 2, 3, 6 and 8) could be detected in any of the skin-prick extracts (fig. 3).

### Detection of Cross-Reactive Proteins in Different Fungal Species

**Fig. 4:**
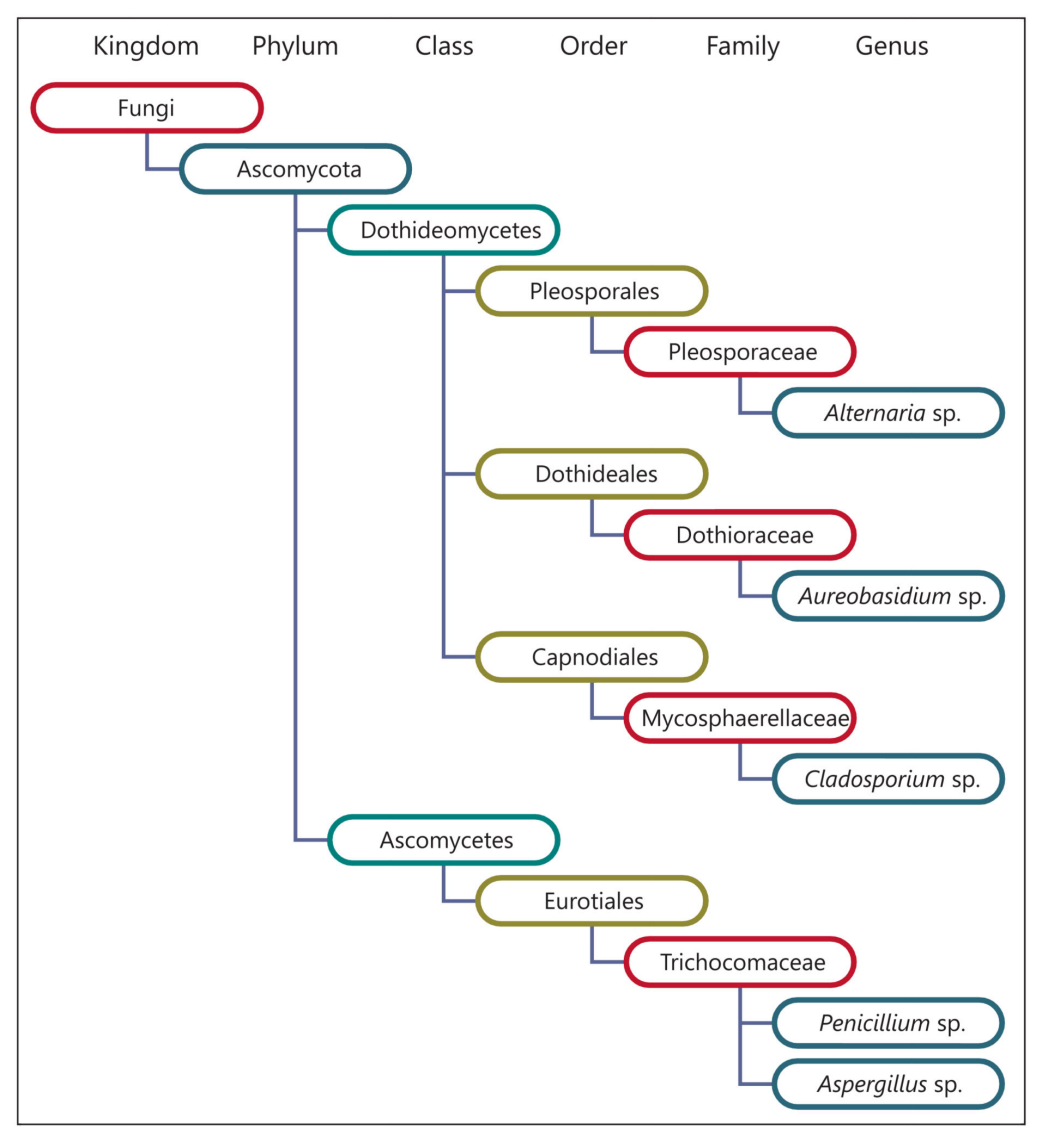
Taxonomic tree of *Alternaria*-related and unrelated mould species.

**Fig. 5:**
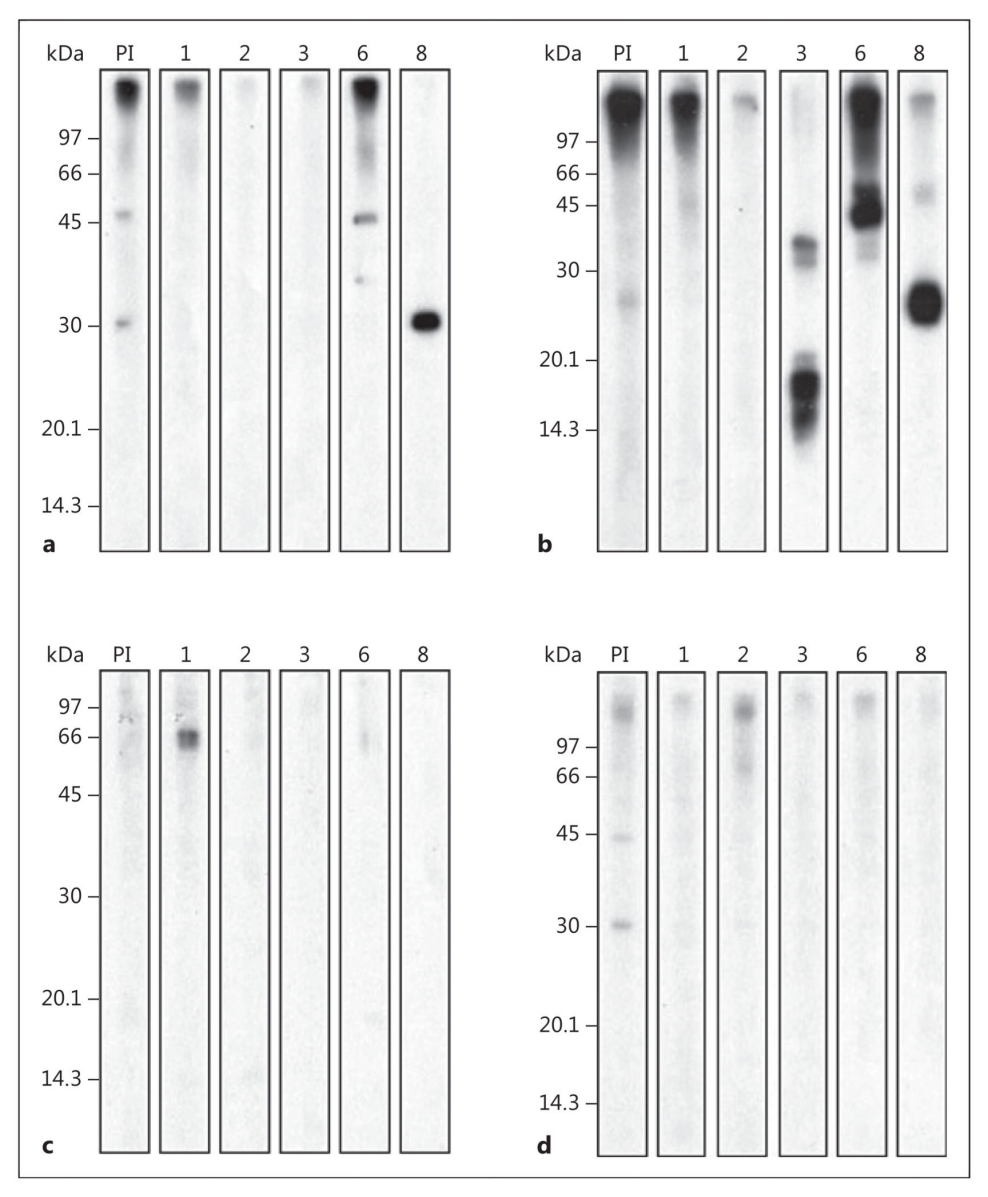
Peptide-specific antibodies recognize homologous proteins in *Alternaria*-related mould species. Nitrocellulose-blotted extracts from *C. herbarum* (**a**), *A. pullulans* (**b**), *A. niger* (**c**) and *P. chrysogenum* (**d**) were exposed to specific rabbit antibodies (lanes 1, 2, 3, 6 and 8: αAlt a 1, αAlt a 2, αAlt a 3, αAlt a 6 and αAlt a 8) or to a mixture of preimmune antisera (lane PI). Molecular weights are indicated in the left margin.

Figure 4 provides an overview of the taxonomical relationship of different fungi; the different genera (*Alternaria*, *Aureobasidium*, *Cladosporium*, *Penicillium* and *Aspergillus*) are listed on the right. Using allergen-specific antibodies, we searched for homologous allergens in the different mould species (fig. 4). Nitrocellulose-blotted extracts of *C. herbarum*, *A. pullulans*, *A. niger* and *P. chrysogenum* were probed with the allergen-specific antibodies and control antibodies (fig. 5a–d). None of the major allergens (i.e. Alt a 1 and Alt a 2) could be detected in any of the 4 mould strains. Bands reacting with the antibodies raised against Alt a 6 and Alt a 8 were found in *C. herbarum* and *A. pullulans*. In the latter, a positive signal was also obtained with the anti-Alt a 3 antibodies (fig. 5a–d). Sequence comparisons of the Alt a 6 peptide with the sequences of the homologous enolases in *C. herbarum* (Cla h 6) and *A. fumigatus* (Asp f 22) revealed an identity of 96.3 and 88.9%, respectively. The Alt a 8 peptide showed a 85.7% sequence identity with the *C. herbarum* allergen Cla h 8 (data not shown). No enolase-like and mannitol dehydrogenase-like proteins have been identified in *A. niger* and *P. chrysogenum* and allergens from *A. pullulans* have so far not been identified.

## Discussion

In this study, we produced specific antibodies against regions with predicted high antigenicity of important *A. alternata* allergens (Alt a 1, Alt a 2, Alt a 3, Alt a 6 and Alt a 8). For this purpose, synthetic peptides of between 26 and 28 amino acids were selected from regions of the allergens with predicted high surface probability. This methodology has been used earlier to predict antigenic peptides from Alt a 1 [25]. The advantage of the peptide-specific antibody probes is that they specifically target the protein backbone of the allergens and do not react with cross-reactive carbohydrate structures which are present in many natural allergen sources.

Using these antibody probes, we first investigated interstrain variablities of allergen expression in 4 *A. Alternata* strains. Interestingly, we found that certain strains expressed very high levels of the major allergen, Alt a 1 (e.g. CBS 103.33) whereas other strains produced larger amounts of Alt a 8 (e.g. CBS 795.72) under given culture conditions. When we tested the strains in different media and considered different time periods in culture, we defined conditions (i.e. combinations of strains and culture conditions) yielding a high allergen expression and conditions that resulted in almost no allergen expression. Interestingly, commercial *Alternaria* extracts, which are usually prepared from liquid cultures, contained only Alt a 1, and no Alt a 3, 6 or 8 could be detected in these extracts (fig. 3). By contrast, extracts prepared from *Alternaria* cultured on solid-phase plates also contained Alt a 3, 6 and 8; this may be due to the fact that solid-phase growth more closely mimics natural mould growth than liquid cultures. We also found that allergic patients showed different IgE reactivity profiles to *Alternaria* extracts obtained under different culture conditions, indicating that the results obtained with the antibody probes may be clinically relevant (fig. 1a).

The antibody probes should therefore be useful to optimize culture conditions yielding high expression of relevant *Alternaria* allergens such as Alt a 1, 3, 6 and 8.

The only allergen which was not detectable in any of the strains tested, even under varying culture conditions, was Alt a 2. In fact, data regarding the relevance of Alt a 2 as an allergen are conflicting. In one study, 61% (16/26) *A. alternata*-sensitized patients recognized rAlt a 2 in a dot-blot analysis, suggesting that it represents a major allergen [29]. However, other investigators could not detect any IgE reactivity to rAlt a 2 in 42 patients allergic to *A. alternata* [30]. In fact, when we compared the published Alt a 2 sequence with the sequences deposited in the NIH databases, we found a 98% sequence identity over the complete Alt a 2 sequence with an *Escherichia coli* protein but no significant sequence homology with other mould proteins. It is therefore possible that the Alt a 2 sequence is not derived from *Alternaria*, which may be the reason why Alt a 2 was not recognized by the IUIS Allergen Nomenclature Subcommittee and is not registered in the allergen database.

The specific antibodies were also used to study the composition of diagnostic *Alternaria* skin-prick solutions from 5 manufacturers. Interestingly, only varying amounts of Alt a 1, but none of the other allergens, was detectable in the diagnostic extracts. The antibodies raised by us should therefore also be useful to characterize natural *Alternaria* extracts for diagnostic and therapeutic purposes.

Finally, we used the antibody probes to screen extracts from 3 other mould species (*C. herbarum*, *A. niger* and *P. chrysogenum*), commonly associated with the development of mould allergy, for the presence of cross-reactive antigens. *A. niger* was used as a representative *Aspergillus* strain because *A. fumigatus*, the most common allergenic *Aspergillus* strain, could not be cultivated in our laboratory. The black yeast *A. pullulans* was also included because it is widely distributed in indoor environments, especially in sauna and bathroom facilities but also in potted plants. Furthermore, it is closely related to the fungus *Alternaria* and has been suggested as a potential elicitor for allergic asthma [31, 32]. Cross-reactive proteins (Alt a 6, Alt a 8 and Alt a 3) were detected in the more closely related mould species *C. herbarum* and *A. pullulans*, which belong to the same class (Dothideomycetes) as *A. alternata*. Indeed, cross-reactivity of the *Alternaria* enolase (Alt a 6) and mannitol dehydrogenase (MtDH, Alt a 8) has already been reported for Cla h 6 and Cla h 8 [33, 34]. So far, no allergens from *A. pullulans* have been identified, but our data indicate that cross-reactive proteins of enolase, MtDH and heat-shock protein occur in this yeast.

In summary, we generated peptide-specific antibodies for important *A. alternata* allergens and demonstrated that they were useful tools to establish culture conditions that allowed high allergen expression, to characterize diagnostic mould extracts and to detect cross-reactive antigens in other mould species. Furthermore, these antibodies may be useful tools for the development of allergen-screening assays and for a more precise estimation of the health risks in households and the workplace. Future applications may include the detection of allergens in environmental samples and the quality control of diagnostic and therapeutic allergen extracts.

## Figures and Tables

**Table 1:** Detection of *Alternaria* allergens in different *A. alternata* strains under different culture conditions

	2 weeks					4 weeks				
	1	2	3	6	8	1	2	3	6	8
CBS 103.33										
Czapek-Dox	+	–	+	+	+	–	–	+	+	+
Malt extract	+	–	+	+	–	+	–	–	–	–
Sabouraud	+	–	–	–	–	+	–	–	–	–
CBS 795.72										
Czapek-Dox	+	–	+	+	+	+	–	+	+	+
Malt extract	+	–	+	+	+	+	–	+	+	+
Sabouraud	+	–	+	+	+	+	–	–	+	–
ATCC 46582										
Czapek-Dox	+	–	–	+	+	+	–	–	+	+
Malt extract	+	–	+	+	+	+	–	–	–	+
Sabouraud	+	–	+	+	+	+	–	–	–	–
ATCC 96154										
Czapek-Dox	–	–	+	+	+	–	–	+	+	+
Malt extract	–	–	+	+	+	–	–	–	–	–
Sabouraud	–	–	–	–	–	–	–	–	–	–

Four *A. alternata* strains (CBS 103.33, CBS 795.72, ATCC 46582 and ATCC 96154) were grown for 2 or 4 weeks on 3 different culture media (Czapek-Dox, Malt extract and Sabouraud) and screened with rabbit antibodies specific for Alt a 1 (1), Alt a 2 (2), Alt a 3 (3), Alt a 6 (6) or Alt a 8 (8). + = Detectable; − = not detectable.
